# 术后淋巴结阴性肺浸润性黏液腺癌患者的预后预测模型

**DOI:** 10.3779/j.issn.1009-3419.2024.106.02

**Published:** 2024-01-20

**Authors:** Zheng WANG, Jinxian HE, Haibo SHEN, Xiaohan CHEN, Chengbin LIN, Hongyan YU, Jiajun GAO, Xianneng HE, Weiyu SHEN

**Affiliations:** ^1^315211 宁波，宁波大学医学部（王正，陈啸晗，高家俊，何贤能）; ^1^Health Science Center, Ningbo University, Ningbo 315211, China; ^2^315048 宁波，宁波大学附属李惠利医院（贺靳贤，林城斌，俞红艳，沈韦羽）; ^2^The Affiliated Lihuili Hospital of Ningbo University, Ningbo 315048, China; ^3^315099 宁波，宁波市第二医院（沈海波）; ^3^Ningbo No.2 Hospital, Ningbo 315099, China

**Keywords:** 肺肿瘤, 肺浸润性黏液腺癌, 淋巴结阴性, 预后, 预测模型, Lung neoplasms, Invasive mucinous adenocarcinoma, Node-negative, Prognosis, Prediction model

## Abstract

**背景与目的** 肺浸润性黏液腺癌（invasive mucinous adenocarcinoma of the lung, IMA）是肺腺癌中一种少见且特殊的类型，该类肿瘤的特点往往是少有淋巴结转移，因此对于该类肿瘤的预后评估依靠现有的肿瘤原发灶-淋巴结-转移（tumor-node-metastasis, TNM）分期存在困难。本研究的目的是构建列线图来预测术后淋巴结阴性的IMA患者的预后。 **方法** 根据纳入标准和排除标准，回顾性分析2012年7月至2017年5月宁波大学附属李惠利医院（训练队列，n=78）和宁波市第二医院（验证队列，n=66）胸外科收治的术后病理为淋巴结阴性的IMA患者的资料，分析训练队列的临床病理特征的预后价值并建立预后预测模型，并对模型性能进行评价，最后将验证队列的数据代入进行外部验证。 **结果** 单因素分析显示肺炎型、较大的肿块、包含黏液和非黏液成分的混合型、较高的总分期是5年无进展生存期（progression-free survival, PFS）及总生存期（overall survival, OS）的影响因素。多因素分析进一步表明，影像学分型、肿块大小、黏液成分是5年PFS及OS的独立预后因素。5年PFS率和OS率分别为62.82%和75.64%，亚组的生存分析显示，肺炎型和包含黏液和非黏液成分的混合型IMA患者的5年PFS及OS分别明显低于孤立型和纯黏液型IMA患者。5年PFS和OS的Harrell’s C指数分别为0.815（95%CI: 0.741-0.889）和0.767（95%CI: 0.669-0.865），这两个模型的校准曲线及决策曲线分析（decision curve analysis, DCA）在两个队列中显示出良好的预测性能。 **结论** 本次基于临床病理特征构建的列线图在一定程度上可以作为IMA切除术后淋巴结阴性患者的一种有效预后预测工具。

## Abstract (en)

**Background and objective** Invasive mucinous adenocarcinoma (IMA) was a rare and specific type of lung adenocarcinoma, which was often characterized by fewer lymphatic metastases. Therefore, it was difficult to evaluate the prognosis of these tumors based on the existing tumor-node-metastasis (TNM) staging. So, this study aimed to develop Nomograms to predict outcomes of patients with pathologic N0 in resected IMA. **Methods** According to the inclusion criteria and exclusion criteria, IMA patients with pathologic N0 in The Affiliated Lihuili Hospital of Ningbo University (training cohort, n=78) and Ningbo No.2 Hospital (validation cohort, n=66) were reviewed between July 2012 and May 2017. The prognostic value of the clinicopathological features in the training cohort was analyzed and prognostic prediction models were established, and the performances of models were evaluated. Finally, the validation cohort data was put in for external validation. **Results** Univariate analysis showed that pneumonic type, larger tumor size, mixed mucinous/non-mucinous component, and higher overall stage were significant influence factors of 5-year progression-free survival (PFS) and overall survival (OS). Multivariate analysis further indicated that type of imaging, tumor size, mucinous component were the independent prognostic factors for poor 5-year PFS and OS. Moreover, the 5-year PFS and OS rates were 62.82% and 75.64%, respectively. In subgroups, the survival analysis also showed that the pneumonic type and mixed mucinous/non-mucinous patients had significantly poorer 5-year PFS and OS compared with solitary type and pure mucinous patients, respectively. The C-index of Nomograms with 5-year PFS and OS were 0.815 (95%CI: 0.741-0.889) and 0.767 (95%CI: 0.669-0.865). The calibration curve and decision curve analysis (DCA) of both models showed good predictive performances in both cohorts. **Conclusion** The Nomograms based on clinicopathological characteristics in a certain extent, can be used as an effective prognostic tool for patients with pathologic N0 after IMA resection.

原发性的肺黏液腺癌包括原位黏液腺癌、微浸润黏液腺癌、浸润性黏液腺癌（invasive mucinous adenocarcinoma of the lung, IMA）和胶样腺癌等。本文主要关注IMA。根据目前世界卫生组织（World Health Organization, WHO）的肺肿瘤分类，IMA是肺腺癌的一种亚型，约占肺腺癌的5%。IMA的病理形态以柱状或杯状细胞为特征，细胞质顶部有丰富的黏蛋白（mucins），细胞核位于基底部。IMA病理上常表现为跳跃性病变（skip lesions）^[1,2]^。与其他亚型的肺腺癌相比，IMA具有明显不同的遗传、临床病理和放射学特征，因此IMA的预后不像其他亚型的肺腺癌的预后那样较为典型。由于IMA发病率低，对IMA的临床病理、影像学和预后特征研究甚少，因此，在既往的研究中IMA的预后往往相互矛盾。一些研究报道^[3,4]^认为，与非黏液性肺腺癌相比，IMA预后中等；另一些报告^[5,6]^则认为IMA的预后会更差，这些表明IMA可能是一种高度异质性的肿瘤。

淋巴结转移在肺腺癌的预后中起着至关重要的作用，因为有无淋巴结转移，患者的生存结果会存在显著差异^[7]^。在既往的研究^[8⇓-10]^中发现大部分IMA患者没有淋巴结转移。其中，Lee等^[3]^还特别强调IMA与较低的淋巴结转移率相关。此外，在本研究组的临床工作中，也碰到过一些肿块较大的IMA患者，但其术后病理也往往为淋巴结阴性。

鉴于IMA患者的术后预后仍然不清楚且病理常常为淋巴结阴性，我们认为有必要对这部分患者进行预后评估，并建立相关的列线图（列线图通过揭示肿瘤预后的相关特征来量化风险），旨在更好地反映现实世界中大多数IMA患者的生存状况，为临床医生评估IMA的预后提供一些参考依据。

## 1 资料与方法

### 1.1 临床资料

回顾性分析2012年7月至2017年5月宁波大学附属李惠利医院胸外科收治的术后病理为淋巴结阴性的IMA患者的资料，共确认78例；宁波市第二医院胸外科收治的术后病理为淋巴结阴性的IMA患者的资料，共确认66例。纳入标准：术前有完整的临床和影像学数据，且经术后病理证实为IMA的患者。排除标准：（1）被诊断为原位黏液腺癌或微浸润黏液腺癌的患者；（2）有其他恶性肿瘤个人史的患者；（3）伴淋巴结转移及远处转移的患者；（4）缺乏完整术后随访记录的患者。涉及到本院患者的研究，已获得宁波大学附属李惠利医院伦理委员会的批准（编号：KY2019PJ058）。

根据美国癌症联合委员会（American Joint Committee on Cancer, AJCC）第8版肿瘤原发灶-淋巴结-转移（tumor-node-metastasis, TNM）分期标准确定肿瘤临床分期。通过电话随访和门诊随访获得生存结局（包括生存或死亡时间及肿瘤复发时间）。无进展生存期（progression-free survival, PFS）是指从手术日到第一次肺癌相关复发或最后一次随访的时间。术后复发主要通过影像学检查确定，包括胸部计算机断层扫描（computed tomography, CT）、淋巴结B超等，并不一定需要组织学证实。总生存期（overall survival, OS）是指从手术日到死亡或最后一次随访的时间。随访时间为每6个月随访一次。训练队列末次随访时间为2022年5月，验证队列末次随访时间为2023年10月。

### 1.2 影像评估

根据术前胸部CT的主要表现形式，78例IMA定性分为两组：孤立型（定义为孤立的结节或肿块）和肺炎型（定义为无明确形状的实变影，沿肺叶或肺段分布）。其他影像学特征，如受累肺叶的位置和肿瘤大小，也被回顾性评估。在随访中胸部CT的影像学表现被认为是确认患者有无复发的重要证据之一。

### 1.3 病理分析

切除的肿瘤标本和淋巴结标本由宁波市临床病理诊断中心有经验的病理科医师评估。IMA的病理诊断是根据2021年WHO肺肿瘤的组织学分类确认的。IMA的特征性病理表现为柱状或杯状细胞结构，细胞核位于基底，细胞质内黏蛋白丰富。根据黏液成分占比，IMA可分为纯黏液型（≥90%浸润性黏液成分，贴壁型为主）和混合型（≥10%浸润性非黏液成分）两组。

### 1.4 统计分析

采用R 4.2.1软件以及SPSS 22.0软件统计数据和分析，采用单因素Cox回归分析初步筛选影响5年PFS和OS的变量，计算风险比（hazard ratio, HR）和95%置信区间（confidence interval, CI）。单因素Cox分析中P<0.05的变量进一步纳入多因素Cox回归分析当中，得到独立的预后因素，并在此基础上构建一个5年PFS的列线图和一个5年OS的列线图。采用Harrell’s C指数、校正曲线和决策曲线分析（decision curve analysis, DCA）对列线图的预后预测模型进行准确的评估及验证。在亚组中分析，采用Kaplan-Meier生存曲线和Log-rank检验评估影像学分型和黏液成分占比的5年生存差异。所有统计检验均为双侧检验， P<0.05为差异具有统计学意义。

## 2 结果

### 2.1 临床病理特征及预后

78例IMA术后患者的临床病理特征见表1，包括46例女性（59.0%）和32例男性（41.0%），中位年龄为60岁，年龄范围为36-81岁。病理标本的肿瘤大小中位数为2.0 cm（范围：0.6-9.0 cm）。孤立型66例，肺炎型12例。单纯黏液型68例，混合型10例。亚肺叶切除15例（19.2%），肺叶切除63例（80.8%）。30例（38.5%）患者术后接受辅助化疗。根据第8版TNM分期系统，65例（83.3%）患者为I期，11例（14.1%）患者为II期，2例（2.6%）患者为III期。中位随访时间为89.80个月（随访时间为8.13-119.70个月）。随访结束时，死亡24例（30.8%），肿瘤进展29例（37.2%），5年PFS率和OS率分别为62.82%和75.64%（图1）。在外部验证队列中，共66例IMA术后患者被纳入，包括36例女性（54.5%）、30例男性（45.5%），中位年龄61岁，年龄范围为38-83岁。肿瘤大小的中位数为1.6 cm（范围：0.2-10.0 cm）。孤立型56例，肺炎型10例。单纯黏液型49例，混合型17例。亚肺叶切除7例（10.6%），肺叶切除59例（89.4%）。56例（84.8%）患者为I期，10例（15.2%）患者为II期。中位随访时间为92.90个月（随访时间为8.17-121.60个月）。随访结束时，死亡21例（31.8%），肿瘤进展28例（42.4%），5年PFS率和OS率分别为69.70%和78.79%。

**表 1:** 78例肺黏液腺癌患者临床病理特征的单因素分析

Characteristics	n	PFS			OS	
		HR (95%CI)	P		HR (95%CI)	P
Age, median (range) (yr)	60 (36-81)					
<60 vs ≥60	34 (43.6%)/44 (56.4%)	1.136 (0.542-2.380)	0.735		0.736 (0.330-1.639)	0.453
Gender						
Female vs Male	46 (59.0%)/32 (41.0%)	1.028 (0.491-2.152)	0.942		1.576 (0.708-3.511)	0.265
Tumor location						
Middle, lower vs Upper	43 (55.1%)/35 (44.9%)	0.982 (0.472-2.041)	0.961		1.080 (0.483-2.414)	0.851
Type of imaging						
Pneumonic type vs Solitary type	12 (15.4%)/66 (84.6%)	0.263 (0.118-0.585)	0.001		0.254 (0.108-0.598)	0.002
Type of surgery						
Sublobar vs Lobe	15 (19.2%)/63 (80.8%)	0.920 (0.375-2.260)	0.856		1.314 (0.449-3.847)	0.618
Tumor size, median (range) (cm)	2.0 (0.6-9.0)	1.355 (1.128-1.629)	0.001		1.426 (1.174-1.733)	<0.001
Mucinous component						
Mixed vs Pure	10 (12.8%)/68 (87.2%)	0.124 (0.055-0.279)	<0.001		0.320 (0.126-0.813)	0.017
Pleural visceral invasion						
No vs Yes	67 (85.9%)/11 (14.1%)	0.989 (0.344-2.843)	0.984		1.391 (0.475-4.076)	0.547
Vascular invasion						
No vs Yes	75 (96.2%)/3 (3.8%)	0.046 (0.000-90.879)	0.427		0.046 (0.000-212.859)	0.475
Nerve invasion						
No vs Yes	76 (97.4%)/2 (2.6%)	1.469 (0.200-10.817)	0.706		1.946 (0.262-14.444)	0.515
Pathological stage						
I vs II+III	65 (83.3%)/13 (16.7%)	2.788 (1.231-6.313)	0.014		2.725 (1.128-6.583)	0.026
Adjuvant chemotherapy						
No vs Yes	48 (61.5%)/30 (38.5%)	0.752 (0.342-1.652)	0.478		0.851 (0.364-1.991)	0.710

IMA: invasive mucinous adenocarcinoma of the lung; PFS: progression-free survival; OS: overall survival; HR: hazard ratio; CI: confidence interval.

**图 1:**
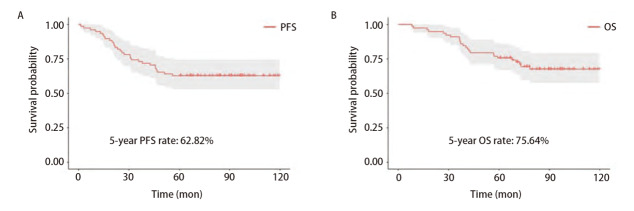
78例IMA患者的生存曲线。 A：5年PFS；B：5年OS。

### 2.2 单因素与多因素Cox回归分析

对78例IMA术后患者进行单因素Cox回归分析发现肺炎型（分别为P=0.001和P=0.002）、较大的肿块大小（分别为P=0.001和P<0.001）、混合型（分别为P<0.001和P=0.017）和较晚的总分期（分别为P=0.014和P=0.026）与较差的5年PFS和OS有关。而年龄、性别、肿瘤部位、手术类型、有无胸膜侵犯、有无血管侵犯、有无神经侵犯和术后有无辅助化疗这些因素则与5年PFS和OS没有明显关系（表1）。

将单因素分析中确定的变量用于多因素分析（表2），研究显示影像学分型（P=0.014）、黏液成分（P<0.001）和肿块大小（P=0.002）仍然是5年PFS的独立影响因素；5年OS的多因素Cox比例风险回归模型也显示肺炎型（P=0.017）、混合型（P=0.001）和更大的肿块（P=0.001）是死亡风险增加的独立因素。

**表 2:** 78例IMA患者临床病理特征的多因素分析

Characteristics	n	PFS			OS	
		HR (95%CI)	P		HR (95%CI)	P
Type of imaging (Pneumonic type vs Solitary type)	12 (15.4%)/66 (84.6%)	0.352 (0.153-0.812)	0.014		0.333 (0.135-0.824)	0.017
Mucinous component (Mixed vs Pure)	10 (12.8%)/68 (87.2%)	0.096 (0.041-0.228)	<0.001		0.182 (0.065-0.507)	0.001
Tumor size, median (range) (cm)	2.0 (0.6-9.0)	1.401 (1.134-1.730)	0.002		1.463 (1.169-1.831)	0.001

### 2.3 亚组生存分析

进行亚组的生存分析（图2）显示，78例患者中包括12例肺炎型（15.4%）和66例孤立型（84.6%），肺炎型和孤立型患者的5年PFS和OS差异均有统计学意义（分别为P<0.001和P=0.001），孤立型患者的5年PFS和OS均要长于肺炎型患者。78例患者根据黏液成分占比分为混合型患者组（10例）和纯黏液型患者组（68例），两组的5年PFS（P<0.001）和OS（P=0.012）的差异有统计学意义。

**图 2:**
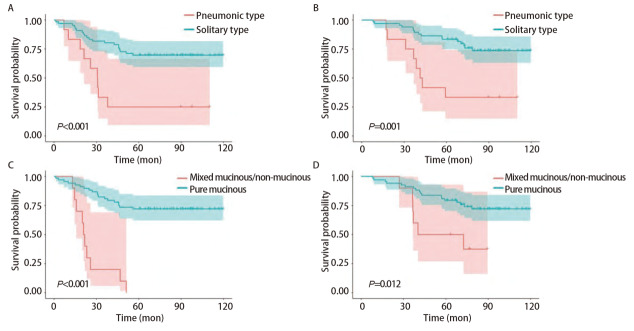
采用Kaplan-Meier法，根据影像学分型分析IMA患者的5年PFS（A）和OS（B）；根据黏液成分占比分析IMA患者的5年PFS（C）和OS（D）。

### 2.4 预测模型的建立及验证

为了提供对临床有用的预后预测模型，整合多因素回归分析结果后使用与5年PFS和OS相关的危险因素来构建列线图，均包含3个独立危险因素（影像学分型、黏液成分占比和肿块大小）。通过各变量对应其线条的长度汇总各变量的得分，其总得分对应的生存概率即为该患者预测的5年PFS和OS。这两个列线图中显示黏液成分是影响预后的最重要因素，而非影像学分型（图3）。其次，这两个预后模型的Harrell’s C指数分别为0.815（95%CI: 0.741-0.889）和0.767（95%CI: 0.669-0.865），预测精度较好。校正曲线显示PFS和OS的5年生存概率预测与实际吻合，偏差轻微；DCA曲线则通过量化净收益来评估预测模型的预测能力。这两个预测模型的DCA曲线均在右上象限，说明模型具有较为精确的预测能力（图4）。在外部验证队列当中，5年PFS和OS的Harrell’s C指数分别为0.767（95%CI: 0.681-0.853）和0.731（95%CI: 0.627-0.835），校准曲线和DCA曲线也表明两个预测模型与实际观测结果较为一致（图4），新开发的列线图可以为术后淋巴结阴性的IMA患者的生存提供一些参考价值。

**图 3:**
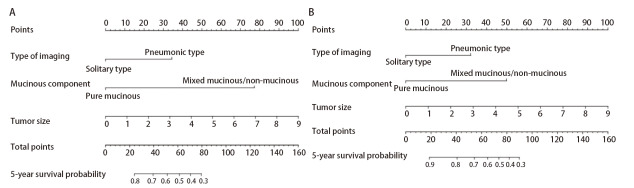
预测IMA患者生存的预后列线图。 A：5年PFS；B：5年OS。

**图 4:**
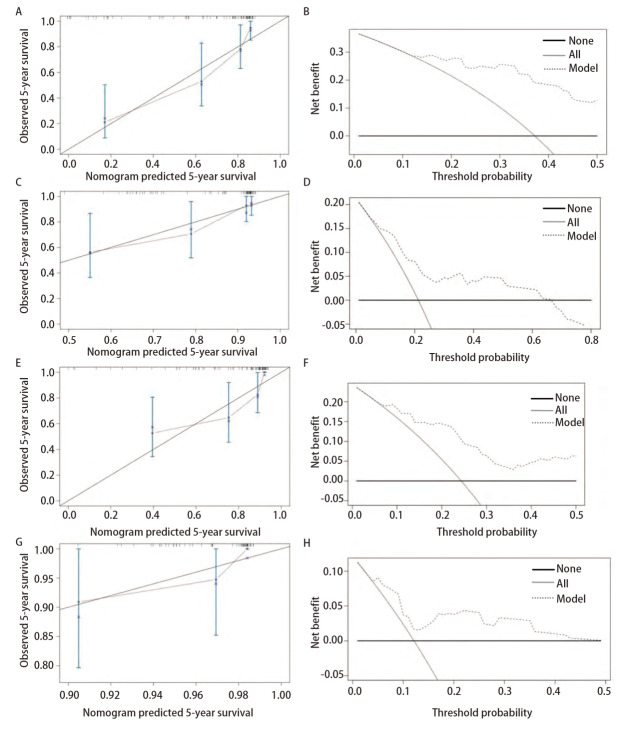
预测模型的构建及验证。 训练队列：用于预测IMA患者5年PFS的校准曲线（A）和DCA曲线（B）；验证队列：用于预测IMA患者5年PFS的校准曲线（C）和DCA曲线（D）。训练队列：用于预测IMA患者5年OS的校准曲线（E）和DCA曲线（F）；验证队列：用于预测IMA患者5年OS的校准曲线（G）和DCA曲线（H）。

## 3 讨论

肺黏液腺癌的概念最早是Kish等^[11]^在1989年的一份病例报告中提出的。从那时起，肺黏液腺癌的定义在过去的几十年里一直在修订^[2,12]^。2015年WHO更新了肺腺癌的分类，肺黏液腺癌被分为原位黏液腺癌、微浸润性黏液腺癌、IMA和胶样腺癌等，其中IMA最常见^[1]^。目前，2021年WHO肺腺癌分类则继续采用之前对IMA的解释，其肿瘤细胞呈杯状或柱状，细胞质内黏液丰富，核小，常被推向细胞一侧，周围肺泡腔也常充满黏液。IMA常呈贴壁生长模式，同时也可以检测到侵袭性成分，如腺泡、乳头状、微乳头状或实体型等。当侵袭性非黏液成分占肿瘤≥10%时，诊断应为混合型IMA。通过基因组学分析，IMA与其他肺腺癌存在不同类型的鼠类肉瘤病毒癌基因同源物（Kirsten rat sarcoma viral oncogene homolog, KRAS）基因突变，IMA中最常见的类型是G12D和G12V突变，而其他肺腺癌中最常见的是G12C突变。G12D和G12V突变在结直肠癌和胰腺癌中也很常见，这表明IMA可能在生物学上与它们更相似^[13⇓-15]^。虽然表皮生长因子受体（epidermal growth factor receptor, EGFR）基因突变在肺腺癌中很常见，但在IMA中非常罕见^[16]^。因此，在目前的实践中，多数IMA患者接受EGFR酪氨酸激酶抑制剂（tyrosine kinase inhibitor, TKI）靶向治疗的机会很低。本研究显示是否接受化疗在5年PFS和OS上没有明显差异。在对I期IMA患者的研究中，Luo等^[17]^得出结论，IMA患者的术后辅助化疗没有显著的生存获益。Cha等^[18]^在对IV期IMA患者的研究中也得出结论，与未治疗的IMA患者相比，接受铂类化疗的IMA患者OS没有改善，因此铂类常规化疗可能对IMA患者没有益处。所以对于术后或晚期IMA患者，化疗无用以及缺少靶向治疗是我们临床工作的难点，仍需要寻找新的化疗方法或研究新的靶向药。此外，现有文献^[3⇓⇓-6]^对IMA与其他肺腺癌在预后方面的报道是矛盾的，有些研究^[3,5,6]^认为IMA预后与中分化腺癌相当，甚至更差；也有一些研究^[4]^认为预后较好。这些报告了相对较好预后的样本都是在亚洲人群中。本研究中，在排除了淋巴结转移这个重要因素后，得出了5年PFS和OS率分别为62.82%和75.64%。

在本研究中，女性患者占IMA病例的大多数（59%）。IMA的女性发病率与其他研究^[19]^一致（53.6%-58.6%），表明IMA可能以女性为主。我们的研究显示43例肿瘤发生在中下叶，35例肿瘤发生在上叶，差异不大。然而，根据以往的报道^[18]^，IMA似乎更多地发生在下叶的外周。因此，术前通过支气管镜活检明确诊断IMA往往具有挑战性。胸部CT引导下的肺穿刺活检可以弥补这一缺陷，但其也有其缺点。由于肿瘤周围的肺泡腔通常充满丰富的黏蛋白，有时活检标本可能由黏蛋白池组成，而可能没有刺穿到肿瘤细胞。因此，通过手术切除获得的病理标本进行诊断IMA仍然是最有效的方法。

本研究显示根据影像学分型的肺炎型患者的生存率明显低于孤立型患者。这一结果与之前的几项研究^[20⇓⇓-23]^均一致。其中，Wang等^[21]^有趣地发现，混合了实变影和磨玻璃影的肺炎型IMA比单纯实变型IMA存活时间更长，这可以从病理学上解释为肺炎型是肿瘤呈贴壁生长、具有侵袭性成分以及伴有黏蛋白累积的一种混合模式。然而，Lee等^[3]^分析了62例结节型肿瘤和19例实变型肿瘤的PFS和OS，其差异均无统计学意义（P=0.062和P=0.109）。不同的结果可能是由于研究样本的大小和对影像学分型的定义差异导致。在Kim等^[24]^的研究中，所有肺炎型IMA的复发均为肺实质的转移，其5年PFS与TNM分期为T4期的非小细胞肺癌患者相当。肺炎型IMA的低生存率可能源于IMA的跳跃性病变。从细胞学角度来看，我们推测肿瘤细胞在丰富的肺泡黏液池中自由移动，这些黏液池已经充满了肺泡腔，并逐渐远离原发病灶的肺泡壁上。这可能是气腔播散（spread through air space, STAS）在IMA中比在其他肺腺癌（14.8%-47.6%）和肺鳞癌（30%）中更常见的原因^[25]^。STAS被定义为微乳头簇、实性巢和/或单个癌细胞扩散到主要肿瘤边缘以外的肺实质的气腔中^[26]^。此外，在IMA中还发现了黏蛋白1（mucin 1, MUC1）、黏蛋白5AC（mucin 5AC, MUC5AC）和MUC6等过表达^[27,28]^，特别是MUC5AC在IMA中可以促进癌细胞对凋亡的抵抗，调节先天免疫细胞的活性。这些黏蛋白单独或通过与受体相互作用来介导细胞信号传导，促进癌细胞存活并增加其转移潜力^[29]^。这可能是IMA中STAS发生率较高的细胞层面原因，也解释了IMA中肺炎型的高复发率和较差的预后。值得注意的是，Beck等^[30]^描述的肺炎型IMA有时还会出现空域混浊的自发消退（spontaneous regression of airspace opacities, SRA），这与抗癌药物的使用无关，并不意味着肿瘤正在缩小，相反，这类患者的分期和预后可能更差。因此，在我们的临床实践中，对于肺炎型IMA的诊治除了与难治性的肺炎鉴别外，还需要更加仔细地评估肿瘤的真实反应，以便对肺炎型IMA患者的疗效做出正确的评价。这些发现强调了IMA具有明显的影像学异质性。

本次的另一个亚组分析得出，在5年的PFS和OS中，混合型IMA患者的生存率明显低于纯黏液型IMA患者。其他研究^[6]^也发现了这一点。因此，我们认为混合型IMA中侵袭性成分的存在可能比单纯黏液型表现出更强的生物学行为，细胞学级别越高的侵袭性成分预示着肿瘤的进展和更差的预后。Cha等^[14]^提出了自己的观点，认为混合型IMA中的侵袭性成分可能是由原有的黏液成分转化为高级别成分而来的。本研究的不足是在术后病理资料中没有体现出具体的侵袭性成分。因此，未来有必要对混合型IMA患者含有的具体侵袭性成分进行更深入的研究。

我们的生存预测模型中包含的这些危险因素是易于确定的临床病理变量，它提高了模型的一般适用性，使其成为一个易于使用的评分系统。目前已经有了一些列线图来预测IMA患者的预后。Chen等^[4]^开发了纯黏液型IMA的列线图，他们排除混合型IMA，但经支气管活检确诊的晚期IMA的患者是被纳入的。Zhang等^[31]^基于监测、流行病学和结果数据库（Surveillance, Epidemiology, and End Results, SEER）数据库和大型外部队列开发并验证了手术切除的IMA列线图，但其研究中使用的肺癌TNM分期系统为第7版。

虽然我们的列线图被证明对于术后病理为淋巴结阴性的IMA患者是一种有效的预测预后模型，但仍然有一些局限性。首先，这是一项回顾性研究，样本量相对较小，不可避免地会产生偏倚；其次，临床病理特征还不包括一些关键的预测因素，如STAS、基因突变状态和混合型IMA中的具体侵袭性成分，因为收集的数据中没有这些信息，无法在本次研究中分析这些变量；第三，对于少部分IMA患者，由于其术中冰冻报告没有明确为IMA，最终病理报告才明确诊断为IMA，因此，对于这部分患者只做了亚肺叶切除术，这也许会导致更高的局部复发率；第四，还需要在更多中心的外部队列中进一步验证列线图。综上所述，影像学分型、黏液成分、肿块大小对5年PFS和OS有影响，我们的列线图对术后淋巴结阴性的IMA患者的5年PFS和OS有一定的参考价值。
